# Depletion of high mobility group box 1(HMGB1) in dendritic cells (DCs) suppresses tumorigenesis and promotes viral clearance

**DOI:** 10.1186/2051-1426-2-S3-P64

**Published:** 2014-11-06

**Authors:** Wenqian Wang, Philip Vernon, Guanqiao Li, Padmavathi Sampath, Thorne Stephen, Xiaoyan Liang, Michael Lotze

**Affiliations:** 1 Departments of Surgery and Immunology, University of Pittsburgh; School of Medicine, Tsinghua University, USA; 2 Naval Medical Research Unit-San Antonio, USA; 3 Departments of Immunology, University of Pittsburgh, PA, USA; 4 Departments of Surgery, University of Pittsburgh, PA, USA; 5 Departments of Surgery, Immunology and Bioengineering, University of Pittsburgh, PA, USA

## Introduction

HMGB1, an evolutionarily ancient and abundant DNA-binding protein within the nucleus, acts as a Damage Associated Molecular Pattern (DAMP) molecule extracellularly to promote immunity. During both cellular stress and immune cell activation, it is translocated into the cytosol and enhances autophagic flux. DCs are critical to both initiating and maintaining T cell adaptive immunity in human cancers. As such, they have formed the basis of many anti-tumor immunotherapies aimed at directing responses to tumor-associated antigens. Vaccinia virus, a large, enveloped virus with dsDNA genome, can infect DCs and inhibit DC maturation. HMGB1 is required for DC functionality and chemotaxis but how HMGB1 regulates DC function in anti-tumor immune response and viral clearance remains unclear.

## Methods

Mice with HMGB1 specifically knocked-out in DCs (DCH) were generated by crossing HMGB1 floxed mice with CD11c Cre mice. For subcutaneous tumor model, wide type (WT), flox/flox and DCH mice were inoculated with 5x10^5 ^Panc02 murine pancreatic cancer cells and 5x10^5 ^MC38 murine colorectal cancer cells. For the bone marrow (BM) transplant model, 8 week old Pdx1-Cre:KrasG12D/+(KC mice) were reconstituted via tail vain injection of 1x10^6 ^freshly isolated BM cells from either DCH or WT after BM progenitor depletion via 1000rad gamma-irradiation. For the virus model, WT and DCH mice were injected with 1x10^7 ^luc+ vaccinia virus.

## Results

While tumors readily grew in both WT and flox/flox mice, the growth was significantly suppressed in DCH mice following Panc02 cancer cell challenge (p < 0.05). The similar inhibitory function was observed in the intrahepatic MC38 tumor model (p < 0.05). In BM transplant experiments, WT to KC chimeras showed both neoplastic ducts and extensive fibrogenesis. KC mice which had received DCH bone marrow exhibited near normal ductal morphology and a significantly diminished occurrence of both low and high grade PanIN lesions (p < 0.05) with reduced levels of myeloid-derived suppressor cells (MDSCs), as well as decreased levels of nominal regulatory T cells (Tregs) in spleen and lower levels of infiltrating cells in pancreas (p < 0.05). Although both WT and DCH mice cleared virus by day 7 following vaccinia virus injection, DCH mice displayed less virus replication and faster viral clearance (p < 0.05). This is associated with increased T cells (p < 0.01) and CD11c+ cells (p < 0.05) within the spleen.

## Conclusion

DC-specific HMGB1 knockout mice not only suppress carcinogenesis and tumor growth but also clear vaccinia viral infection more effectively. This has profound implications for understanding and targeting DCs and HMGB1 for therapy.

